# A retrospective study of 323 total laparoscopic hysterectomy cases for various indications and a case report treating caesarean scar pregnancy

**DOI:** 10.1186/s13256-020-02585-5

**Published:** 2020-12-14

**Authors:** Wataru Isono, Akira Tsuchiya, Michiko Honda, Ako Saito, Hiroko Tsuchiya, Reiko Matsuyama, Akihisa Fujimoto, Masashi Kawamoto, Osamu Nishii

**Affiliations:** 1 Department of Obstetrics and Gynaecology, University Hospital Mizonokuchi, Teikyo University School of Medicine, 5-1-1 Kawasaki, Futago Takatsu-ku, Futago, Kanagawa 213-8507 Japan; 2 Department of Diagnostic Pathology, University Hospital Mizonokuchi, Teikyo University School of Medicine, 5-1-1 Kawasaki, Takatsu-ku, Futago, Kanagawa 213-8507 Japan

**Keywords:** Total laparoscopic hysterectomy, Multivariate analysis, Retrospective study, Surgical complications, Caesarean scar pregnancy

## Abstract

**Background:**

The application of laparoscopic surgeries has been increasing, and various uterine diseases in addition to leiomyoma/adenomyoma have become indications for total laparoscopic hysterectomy (TLH). Therefore, data acquisition and analysis of TLH procedures, including TLH for rare uterine diseases, have become important for improving surgical procedures and patient selection. To determine the prevalence of and risk factors for the occurrence of intraoperative and postoperative complications of TLH, we performed a multivariate analysis of the records in our hospital.

**Methods:**

We retrospectively reviewed the medical records of 323 patients who underwent TLH for the treatment of leiomyoma/adenomyoma (278 cases), low-grade (pre)malignant uterine tumours (40 cases) and other rare uterine diseases (5 cases) from January 1, 2015, to December 31, 2019. Of the rare uterine diseases, one case of caesarean scar pregnancy for which TLH was performed is introduced as a case report. To assess the effects of 11 representative factors, including patient characteristics, uterus and leiomyoma sizes, indications for TLH and others, we performed a multivariate logistic regression analysis.

**Results:**

Among the 323 cases, 20 intraoperative complications and 15 postoperative complications were reported. In the multivariate analysis, “ovarian tumour” and “heavy uterus” were positively associated and “nulliparity” was negatively associated with intraoperative complications. There were no significant risk factors for postoperative complications. The only risk factor for operative complications directly related to the resected uterus was “heavy uterus”. Therefore, we could perform TLH relatively safely for patients with other indications besides leiomyoma/adenomyoma.

**Conclusions:**

Considering the factors detected in this analysis, the indications for TLH may be expanded. Owing to the increase in TLH for indications other than leiomyoma/adenomyoma, a more accurate determination of the treatment approach can be achieved.

## Background

With the widespread application of total laparoscopic hysterectomy (TLH), the number of indications in addition to leiomyoma/adenomyoma has increased [1-3]. The application of TLH for low-grade (pre)malignant uterine tumours as well as other rare uterine diseases, such as ectopic pregnancy, is expected to increase. This is because TLH is associated with relatively less pain and a quicker recovery than conventional laparotomy. Especially in multiparous patients, a relatively low recurrence rate is also beneficial. In our hospital, similarly, the number of TLHs for the treatment of diseases besides leiomyomas/adenomyomas have increased annually [4]. However, improved surgical skills and additional experience are needed. A long operation time is also required [4, 5]. Therefore, data acquisition and analysis of TLH cases, including TLH for rare uterine diseases, are important to improve surgical procedures and patient selection.

Here, we present one rare case in which we performed TLH to successively treat caesarean scar pregnancy to illustrate our approach for treating a rare uterine disease. Simultaneously, we will introduce a proposed improvement for TLH that involves transvaginal morcellation of the uterus without a power device. Since the rates of indications besides leiomyoma/adenomyoma have increased annually with the increase of TLH cases, the safety verification of TLH has become more important. By analysing all the cases in our hospital, we elucidated factors that predicted the possibility of surgical complications.

## Methods

### Data collection and statistical analysis

This study was reviewed and approved by the Human Ethical Committee of the University of Teikyo Hospital (Trial registration number: 20-094). The de-identified medical records of 323 female patients who underwent TLH from June 1, 2015 to December 31, 2019 were reviewed retrospectively. In these cases, bilateral salpingectomy (BS) or BSO was performed during TLH. This study also included 25 cases with concomitant PLA and 19 cases with concomitant LC, including 2 bilateral LCs and 17 unilateral LCs. Among the 323 cases, we extracted representative patients’ characteristics and surgical complications as described below. Statistical analyses were performed using JMP version 12 for Windows (SAS Institute, Inc., Tokyo, Japan) to determine the correlations between patient characteristics and surgical complications. The odds ratios (ORs) and 95% confidence intervals (CIs) were estimated to determine the strengths of the correlations. *P* < 0.05 was considered statistically significant.

### Patient characteristics and analysis methods

The patient characteristics (shown in Table 1) were collected from medical records, and the indications for TLH were classified into three categories, including leiomyoma/adenomyoma, low-grade (pre)malignant uterine tumour, and other rare uterine diseases (shown in Table 2). To identify the risk factors for TLH, we extracted surgical complications and divided them into 2 categories: “intraoperative” (20 cases) and “postoperative” (15 cases) complications (shown in Table 3). We defined “massive bleeding” as a blood loss volume of 500 ml or higher. Among other surgical complications, we regarded bowel injury (1 case) and ureteral injury (1 case) as intraoperative complications and vaginal dehiscence (8 cases) requiring a gauze and/or wound suture, postoperative infection (6 cases) requiring intravenous antibiotics, and postoperative haemorrhage from trocar scars (1 case) as postoperative complications (shown in Table 3). In total, 35 cases of surgical complications were identified; one patient experienced complications in both categories. Since concomitant PLA and LC cases were included, we did not analyse the operation time.

**Table 1:** Patient characteristics

Characteristics	Avg. ± SD (Min.–Max.), number
Age	47.5 ± 6.1 (35–81), *n* = 323
Body mass index (kg/m^2^)	22.9 ± 3.7 (15.9–37.8), *n* = 323
Parity	1.2 ± 1.0 (0–4), *n* = 323
Hospitalization duration (days)	6.4 ± 2.2 (5–40), *n* = 323
Operation time (min)	206.8 ± 57.2 (94–504), *n* = 323
Concomitant procedure	261.5 ± 44.1 (163–359), *n* = 44
No concomitant procedure	198.2 ± 54.2 (94–504), *n* = 279
Blood loss (ml)	132.4 ± 196.8 (0–1500), *n* = 323
Concomitant procedure	164.9 ± 221.3 (0–1150), *n* = 44
No concomitant procedure	127.3 ± 192.6 (0–1500), *n* = 279
Weight of resected uterus (g)	296.7 ± 206.3 (42–1284), *n* = 320
Size of uterus (TVUS) (mm)	73.3 ± 20.1 (29–155), *n* = 323
Size of dominant leiomyoma (MRI) (mm)	62.4 ± 29.2 (15–176), *n* = 276
Size of dominant leiomyoma (TVUS) (mm)	56.2 ± 23.6 (13–130), *n* = 266
Haemoglobin concentration (mIU/ml)	
Before operation	13.0 ± 1.2 (6.6–16.7), *n* = 323
Immediately after operation	11.2 ± 1.3 (6.7–15.0), *n* = 323
Before discharge	11.3 ± 1.3 (7.9–15.5), *n* = 322
Gynaecological surgical history	*n* = 66
Symptomatic patients	*n* = 280
Hypermenorrhoea	*n* = 151
Prolonged menstruation	*n* = 15
Dysmenorrhoea	*n* = 39
Abnormal vaginal bleeding	*n* = 59
Anaemia	*n* = 111
Abdominal compression	*n* = 25
Abdominal pain	*n* = 35
Urination/defecation disorder	*n* = 22
Asymptomatic patients	*n* = 143
Notable findings (on MRI)	
Submucous leiomyoma	*n* = 70
Adenomyoma	*n* = 77
Ovarian tumour	*n* = 40
Autologous blood donation	*n* = 230
Blood transfusion	*n* = 20
Autologous blood transfusion	*n* = 19
Allogeneic blood transfusion	*n* = 1
Annual number of TLHs	
2015	*n* = 24
2016	*n* = 32
2017	*n* = 75
2018	*n* = 106
2019	*n* = 86

Representative patient characteristics obtained from medical records are summarized in this table. For each item, we calculated averages and standard deviations, minimal and maximal values, and count data from medical records. “Size of uterus (TVUS)” and “Size of dominant leiomyoma (TVUS)” were measured on admission. The size of the uterus (according to TVUS) was determined by calculating the average length and width of the uterus. The “size of dominant leiomyoma (MRI)” and “size of dominant leiomyoma (TVUS)” were determined by the maximal diameter of the leiomyoma. In some cases, multiple symptoms occurred in a single patient. In the case of allogeneic blood transfusion, we performed transfusion of 6 IU of red blood cell concentrates

*Avg.* average, *Min.* minimum, *Max* maximum, *SD* standard deviation, *TVUS* transvaginal ultrasound, *MRI* magnetic resonance imaging

**Table 2:** Indications for TLH other than leiomyoma/adenomyoma

Indications	Number
Low-grade (pre)malignanct uterine tumour	40
FIGO stage 1a endometrial carcinoma	28
AEMH	3
CIN	9
CIN 2/3	6
AIS	2
AGC	1
Rare disease	5
Uterine haematoma	1
Caesarean scar pregnancy	1
Cervical ectopic pregnancy	1
Vaginal atresia with molimina	1
Cervical tumour (lymphatic and vessel dilation)	1
Total	45

*FIGO* International Federation of Gynecology and Obstetrics, *AEMH* atypical endometrial hyperplasia, *CIN* cervical intraepithelial neoplasia, *CIS* carcinoma in situ, *AIS* adenocarcinoma in situ, *AGC* atypical glandular cell

**Table 3:** Surgical complications of TLH

Complications	Number
Intraoperative	20
Massive bleeding	18
Bowel injury	1
Ureteral injury	1
Postoperative	15
Vaginal dehiscence	8
Postoperative infection	6
Postoperative haemorrhage from trocar scar	1
Total	35

In this study, surgical complications were divided into 2 categories: intraoperative (20 cases) and postoperative (15 cases) complications

To control for confounding factors, we divided the patients into two groups according to the presence or absence of each factor and performed a multivariate logistic regression analysis. In the 323 patients who underwent TLH, we assessed the influence of the following 11 factors: (1) “advanced age”, defined as an age ≥ 50; (2) “nulliparity”, defined as no previous delivery; (3) “high BMI”, defined as a body mass index (BMI) ≥ 25 (kg/m^2^); (4) “gynaecological surgical history”; (5) “concomitant procedure”, defined as concomitant PLA or LC; (6) “other indications”, defined as TLH for indications besides leiomyoma or adenomyoma; (7) “large leiomyoma”, defined as a dominant leiomyoma ≥ 8 cm as determined by magnetic resonance imaging (MRI); (8) “ovarian tumour”, defined as benign ovarian tumours; (9) “large uterus”, defined as the average uterus length ≥ 10 cm as determined by transvaginal ultrasound (TVUS) immediately before surgery; (10) “heavy uterus”, defined as a resected uterus weighing ≥ 500 g; and (11) “abdominal adhesion”, defined as abdominal adhesion detected by laparoscopic inspection immediately after the start of surgery. The criteria for “large leiomyoma” and “heavy uterus” were determined based on previous reports [6, 7].

### Surgical procedures

**Fig. 1:**
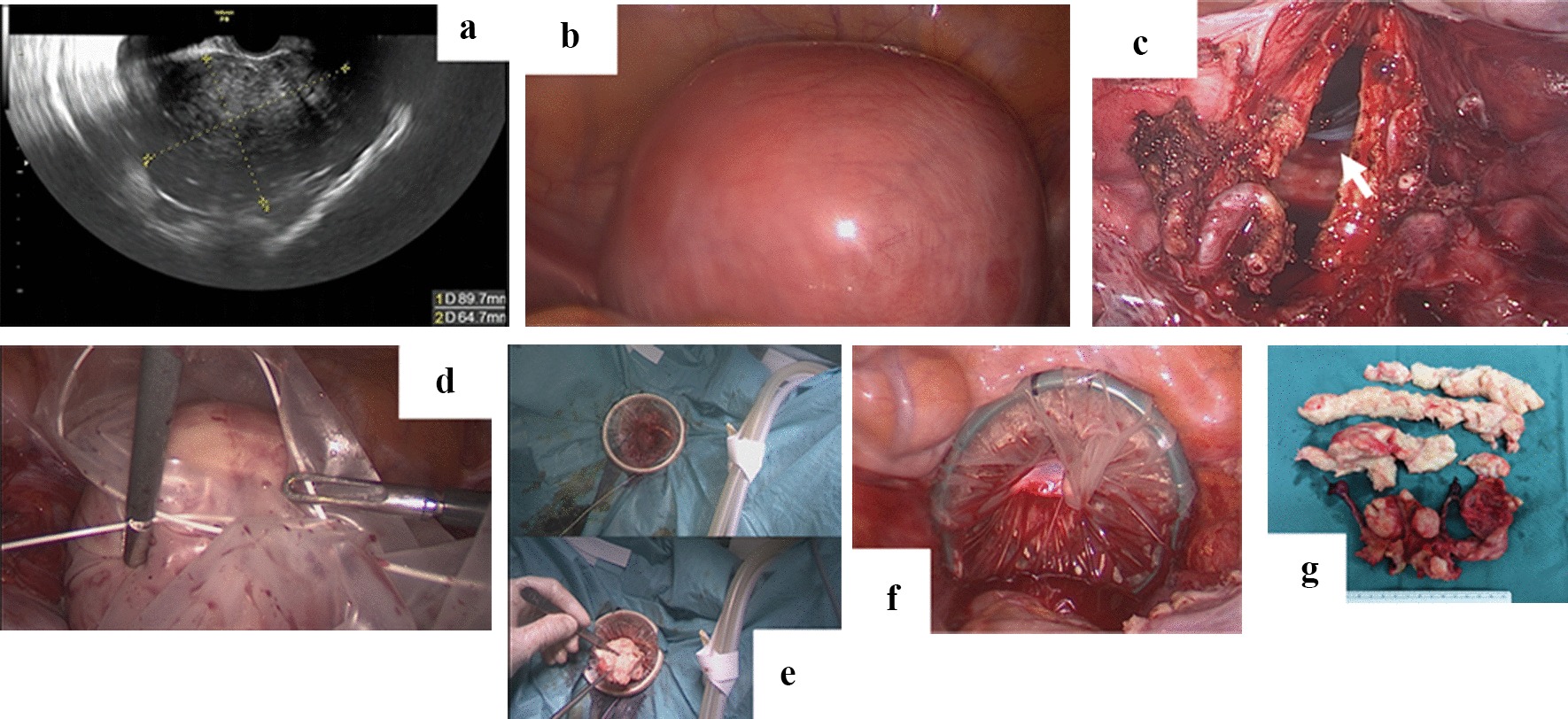
Surgical procedures of transvaginal morcellation of the uterus. This patient who underwent TLH and BS was a 47-year-old woman with multiple uterine leiomyomas. The largest leiomyoma was 8.4 cm in diameter, as detected by magnetic resonance imaging (MRI) during an outpatient examination. The size of the uterus was 90 × 65 mm, as detected by TVUS (**a**). She did not receive administration of GnRHa. She had a history of 0 gravidity and 0 parity, and her BMI was 20.9 kg/m^2^. The operation time was 134 minutes, and the blood loss volume was 75 ml. Four trocars were placed at the incision sites. The total weight of the uterus and bilateral tubes was 317.8 g. **b** Gross appearance of the uterus at the start of TLH. **c** Circumferential colpotomy using a cylinder-shaped vaginal pipe (arrow). **d** Collected uterus and tubes in 1200-ml MemoBag™. **e**, **f** Visualization of the vaginal wound with the Small Alexis^®^ Wound Protector/Retractor and morcellated uterus (**e** inside, **f** outside). **g** Gross appearance of the uterus and tubes removed during surgery

**Fig. 2:**
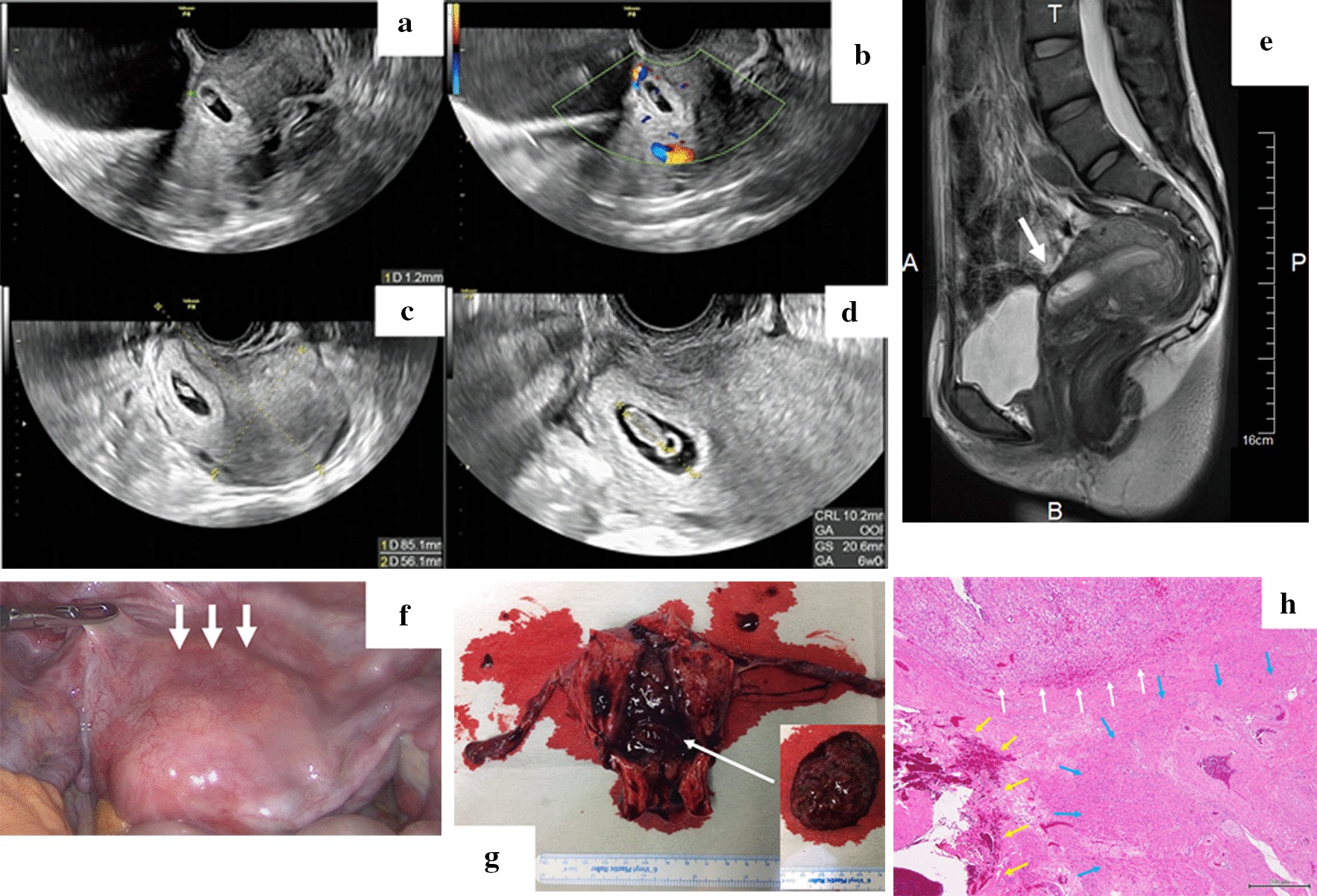
Images and findings of a reported case of caesarean scar pregnancy. **a** Endogenic growth of the GS (measuring 27 × 15 mm) and marked thinning of the uterine cervix wall (measuring 1.9 mm, arrow) were detected by T2-weighted magnetic resonance imaging (MRI) 6 days before surgery (**e**). **b**–**d** The uterus was 85 × 56 mm (**c**), the GS was 20.6 mm with a clear FHB and the CRL was 10.2 mm (**d**) according to TVUS 3 days before surgery. Rich blood flow was detected in the thinned myometrium by colour Doppler sonography 8 days before surgery (**b**). **f** Gross appearance of the uterus at the start of TLH. A clearly thinned blood-vessel rich myometrium was detected in the lower uterine segment (arrow). **g** Gross appearance of the uterus and tubes removed during surgery. Gestational products were detected in the uterine cavity, and their locations were coincident with caesarean scar pregnancy. **h** Pathological findings of caesarean scar pregnancy are indicated by yellow arrows Hematoxylin Eosin (H-E) stain. A normal myometrium was indicated by blue arrows. A clearly thinned blood-vessel rich myometrium was detected. Formation of the decidua and placental villi development was detected near the uterine isthmus (white arrows). Scale bars denote 500 μm

TLH was mainly performed as previously described [8]. The representative characteristics of the procedure are as follows. (1) An umbilical trocar (12 mm) and three lower abdominal trocars (5 mm) were placed in diamond pattern trocar conformation to access the abdominal and pelvic cavities. (2) A harmonic scalpel was used to cut the arteries and ligaments. (3) Bipolar coagulation electrodes were used to control bleeding. (4) A monopolar hook was used for cutting the peritoneum and vaginal cuff. In our hospital, to remove uterus and other tissues from vaginal wounds, we used the following devices: (1) a cylinder-shaped vaginal pipe, which was inserted to check the position of the vaginal fornix prior to circumferential colpotomy (Fig. 1c); (2) an 800-ml or 1200-ml MemoBag™, which was inserted into the pelvic cavity to collect all tissues intraperitoneally (Fig. 1d); and (3) a medium Alexis^®^ wound protector/retractor, which was placed inside the vaginal wound to provide a wide operative view and prevent bleeding from the vaginal wound by applying pressure (Fig. 1e, f). When performing TLH with bilateral salpingo-oophorectomy (BSO) and pelvic lymphadenectomy (PLA) for the treatment of early-stage endometrial carcinoma, one additional upper abdominal trocar (5 mm) was used instead of a uterine manipulator for uterine positioning. In some cases, laparoscopic ovarian cystectomy (LC) was also performed. All operations were performed under the direct supervision of at least one of two physicians (A.F. and O.N.) who are highly skilled laparoscopy specialists accredited by the Japan Society of Gynaecologic and Obstetric Endoscopy and Minimally Invasive Surgery.

### Case presentation

A 35-year-old Japanese woman, gravida 6, para 3, with 2 previous caesarean deliveries, was referred to our hospital at 6 weeks and 3 days gestational age for suspected abnormal placentation. She had a BMI of 25.9 kg/m^2^. Her obstetrical history included one caesarean delivery (8 years ago), one spontaneous vaginal delivery (7 years ago) and a second caesarean delivery (5 years ago). Nine months before visiting our hospital, surgical abortion was performed.

At presentation, TVUS revealed an 18-mm gestational sac (GS), a clear foetal heart-beat (FHB) and a crown-rump length (CRL) of 6.4 mm. The position of the GS was nearer to the internal uterine orifice than normal, and the thickness of the myometrium from the GS to the front of the uterus was 2.9 mm. When performing TVUS again after confirming a urine-filled bladder, the thickness of the myometrium from the GS to the bladder was 1.2 mm (Fig. 2a), and rich blood flow was detected in the thinned myometrium by colour Doppler sonography (Fig. 2b); the uterine cervix and internal uterine orifice could be identified. MRI indicated marked thinning (measuring 1.9 mm) and an abnormally concave outer surface of the anterior uterine cervix wall (Fig. 2e). Endogenic growth of the GS (measuring 27 × 15 mm) was detected in the suspected transverse caesarean scar. The serum human chorionic gonadotrophin (HCG) level was 37886.7 mIU/ml. The haemoglobin concentration was 14.1 g/dl, and no abnormal parameters were observed. After the imaging was performed and caesarean scar pregnancy was diagnosed, various treatment options, including systemic/local injection of methotrexate (MTX) with/without hysteroscopic or laparoscopic surgery, were explained to the patient and her husband. They selected laparoscopic hysterectomy because this method has a short treatment period and is associated with a very low recurrence rate.

Laparoscopic hysterectomy and BS were performed under general anaesthesia at 7 weeks and 4 days gestational age. Before surgery, an 85-mm × 56-mm uterus (Fig. 2c), a 20.6-mm GS with a clear FHB and a 10.2-mm CRL were detected by TVUS (Fig. 2d). A soft and enlarged uterus was observed, and a clearly thinned blood-vessel rich myometrium was detected in the lower uterine segment (Fig. 2f). After removing the uterus (weighing 170 g), the locations of other gestational products were confirmed to be indicative of caesarean scar pregnancy (Fig. 2g). Five days after the operation, she was discharged from the hospital with no surgical complications. Approximately 1 month after surgery, the patient visited our hospital with no complaints, and no complications occurred for more than 1 year. The final pathological diagnosis also supported caesarean scar pregnancy since decidua and placental villi development were detected near the uterine isthmus. Thinned myometrium was observed at the location of placental attachment (Fig. 1h).

## Results

### Patient characteristics

As shown in Table 1, the average age, BMI, parity and hospitalization duration of the included patients were 47.5 ± 6.1 (35–81) years old, 22.9 ± 3.7 (15.9–37.8) kg/m^2^, 1.2 ± 1.0 (0–4) parity and 6.4 ± 2.2 (5–40) days, respectively. In total, 184 patients were treated with a gonadotropin-releasing hormone analogue (GnRHa) before surgery. The overall average length of the operation time in the 323 patients was 206.8 ± 57.2 (94–504) min, and the average blood loss volume was 132.4 ± 196.8 (0–1500) ml. When concomitant PLA or LC was performed, the operation time was significantly prolonged, but the blood loss volume was not significantly different. Of the 323 patients, 143 patients had no complaints, but the other 280 patients had various symptoms, such as menstrual disorders and abdominal distension; one patient reported multiple symptoms. According to MRI, the average size of the dominant leiomyoma in 276 patients was 62.4 ± 29.2 (15–176) mm. Other notable findings besides subserosal or intramural leiomyomas were also detected, including submucous leiomyoma (*n* = 70), adenomyoma (*n* = 77) and ovarian tumour (*n *= 40). The average haemoglobin concentrations before the operation, immediately after the operation and before discharge were 13.0 ± 1.2 (6.6–16.7), 11.2 ± 1.3 (6.7–15.0) and 11.3 ± 1.3 (7.9–15.5), respectively. Autologous blood donation was performed in 230 patients, and blood transfusion was performed in 20 patients, including 19 patients who required autologous blood transfusion and one patient who required allogeneic blood transfusion. The annual number of TLH cases increased annually, and approximately 60% of the 323 TLH cases were performed in 2018 or 2019.

### Detailed explanations of indications

We performed 323 TLHs for leiomyoma/adenomyoma (278 cases), low-grade (pre)malignant uterine tumour (40 cases), and other rare uterine diseases (5 cases, Table 2). Among the 278 leiomyoma/adenomyoma patients, 77 patients had adenomyoma, of which 60 patients had both diseases. The 40 (pre)malignant uterine tumour cases included 28 cases of International Federation of Gynaecology and Obstetrics (FIGO) stage 1a endometrial carcinoma, 3 cases of atypical endometrial hyperplasia (AEMH) and 9 cases of cervical intraepithelial neoplasia (CIN). In one case of “cervical ectopic pregnancy”, the patient visited our emergency outpatient clinic for massive vaginal bleeding. Since a sensitive beta hCG qualitative test was negative, abnormal bleeding caused by a uterine cervical carcinoma was suspected. Soon after admission, we performed emergency uterine artery embolization (UEA) and transfusion with 6 IU of red blood cell concentrates. TLH was performed two days later, and during the operation, the transfusion of 10 IU of red blood cell concentrates was needed. In both cases of “uterine haematoma” and “cervical tumour”, due to atypical findings detected on MRI, we could not exclude the possibility of malignant tumours, which were diagnosed as endometrial and cervical carcinomas, respectively. One patient with “vaginal atresia with molimina” had a history of medical treatment with intrauterine devices, and a foreign-body granuloma was detected in the vaginal wall near the squamocolumnar junction of the uterine cervix by pathological examination. The rates of indications besides leiomyoma/adenomyoma mostly increased annually, with 4/24 cases in 2015, 0/32 cases in 2016, 4/75 cases in 2017, 19/106 cases in 2018 and 18/86 cases in 2019. In particular, all 5 rare uterine cases were treated in 2018 or 2019.

### Influential factors of surgical complications

To detect the significant factors affecting the possibility of intraoperative and postoperative complications, multivariate analysis of 11 representative factors was performed (Table 4). According to the analysis, the following three factors affected the rate of intraoperative complications: (1) “nulliparity” (OR = 0.44, *P* < 0.05); (2) “ovarian tumour” (OR = 4.41, *P* < 0.01); and (3) “heavy uterus” (OR = 5.10, *P *< 0.05). “Gynaecological surgical history” (OR = 2.81, *P* = 0.056) and “large leiomyoma” (OR = 4.39, *P* = 0.062) showed a tendency to increase the possibility of intraoperative complications. However, no factor affected the rate of postoperative complications.

**Table 4:** Identification of influential factors for surgical complications

Factors	Number	Intraoperative	*P* value	Postoperative	*P* value
		OR (95% CIs)		OR (95% CIs)	
Advanced age (≥ 50)	80	0.52 (0.15–1.82)	NS	0.45 (0.10–2.06)	NS
Nulliparity	114	0.44 (0.14–1.34)	p < 0.05	1.23 (0.43–3.56)	NS
Higher BMI (≥ 25 kg/m^2^)	82	1.28 (0.48–3.45)	NS	0.20 (0.03–1.55)	NS
Gynaecological surgical history	66	2.81 (1.10–7.20)	NS	0.59 (0.13–2.67)	NS
Concomitant PLA/LC	44	1.64 (0.52–5.17)	NS	1.63 (0.44–6.02)	NS
Other indications	45	1.10 (0.31–3.90)	NS	Impossible to calculate	NS
Large leiomyoma (≥ 8 cm)	77	4.39 (1.75–11.04)	NS	1.17 (0.36–3.79)	NS
Ovarian tumour	40	4.41 (1.64–11.83)	p < 0.01	2.75 (0.83–9.09)	NS
Large uterus (≥ 10 cm)	31	3.55 (1.20–10.55)	NS	1.48 (0.32–6.88)	NS
Heavy uterus (≥ 500 g)	43	5.10 (1.95–13.35)	p < 0.05	0.45 (0.06–3.53)	NS
Abdominal adhesion	138	1.69 (0.68–4.21)	NS	0.66 (0.22–1.97)	NS

A multivariate analysis of 323 patients with TLH was performed to examine the influence of 11 representative factors that were collected from medical records. The number of patients with each factor, the ORs and 95% CIs for occurrence of surgical complications and the p-values are shown in this table. “Nulliparity”, “ovarian tumour” and “heavy uterus” were identified as significant factors for the occurrence of intraoperative complications. No factor was significantly associated with the occurrence of postoperative complications

*BMI* body mass index, *PLA* pelvic lymphadenectomy, *LC* laparoscopic ovarian cystectomy, *OR* odds ratio, *CI* confidence interval, *NS* no significance

## Discussion

To manage the increasing indications for TLH, we must analyse the results of various cases and validate the safety of the procedure for these indications. In our hospital, from 2015 to 2019, approximately one-seventh of all TLHs were performed for indications other than leiomyoma/adenomyoma (45/323 cases). In these 45 cases, we treated 5 rare uterine cases without any serious complications; the rare cases included uterine haematoma, caesarean scar pregnancy, cervical ectopic pregnancy, vaginal atresia and cervical tumour formed by lymphatic and vessel dilation (Table 2). These other indications also included 28 cases of early-stage endometrial carcinoma (International FIGO stage 1a) and 17 cases in which patients received other treatment options in addition to hysterectomy (Table 2). Especially for multiparous patients, increasing attention should be paid to the merits of TLH, as it is a relatively non-invasive procedure and has short treatment period and low recurrence rate. Additionally, when performing TLH for “other indications”, the occurrence of surgical complications did not increase (Table 4). Among these 17 cases, multiparous patients accounted for 13 cases. The rate of multiparity tended to be higher than that in all other cases, though there was no significant difference (76.5%, *n* = 13/17 vs. 64.1%, *n* = 196/306, *P *= 0.30). TLH also had another advantage related to diagnostics. After operations, we directly compared the imaging diagnosis according to MRI to the definitive diagnosis confirmed by pathological findings. These pathological data directly acquired through surgical procedures are valuable since hysterectomy for these indications was generally not selected as a first choice treatment [9-11]. Surgical treatment was probably performed after the failure of conservative treatment. In the presented case, in which we performed TLH to treat caesarean scar pregnancy, the final diagnosis was determined by the pathological findings.

Then, we analysed all 323 cases to identify risk factors for surgical complications, although we selected these items retrospectively, and the results were inevitably limited. Surgical complications were classified into intraoperative and postoperative complications. In the process, we regarded massive blood loss (≥ 500 ml) as an intraoperative complication, but we could not consider the effect of operation time. When compared with published data from large hospitals in Japan [7, 8, 12-20], the average operation time in our hospital seemed long. This was probably caused by the TLH procedure itself, in which transvaginal morcellation of the uterus was performed with only a scalpel, as well as the fact that trainees mainly performed the operations in some cases, though at least one expert participated as an assistant operator and supervised the procedure to prevent problems. To find risk factors for the occurrence of complications based on patient characteristics, a multivariate analysis of 11 factors was performed. Although we did not detect any significant risk factors for postoperative complications, our analysis indicated the effect of a “heavy uterine” and “ovarian tumour” on intraoperative complications. The former factor was simple and is fairly consistent with those of previous reports. The relationship between “ovarian tumour” and intraoperative complications seemed to be somewhat perplexing. However, it is possible that ovarian endometrial cysts coexisting with leiomyoma caused TLH to be difficult, as suggested by the following results: (1) out of 40 cases of “ovarian tumour”, 7 cases of intraoperative complications (all cases were massive blood loss) occurred, and ovarian endometrial cysts were detected in 6 of these cases; (2) in all 7 cases, the indication for TLH was “leiomyoma”; and (3) among the 7 cases, 4 cases of coexisting adenomyoma occurred. When considering these factors, TLH for diseases other than leiomyoma/adenomyoma may be relatively safe.

## Conclusions

This study identified risk factors for surgical complications of TLH, and other indications besides leiomyoma/adenomyoma did not increase the occurrence of complications. By considering these factors, the indications for TLH might be able to be expanded. With the increase in rare indications for TLH, a more accurate determination of the treatment approach can be achieved.

## Data Availability

The authors agree to make all data of this study freely available.

## Abbreviations

TLH: Total laparoscopic hysterectomy
BSO: Bilateral salpingo-oophorectomy
PLA: Pelvic lymphadenectomy
LC: Laparoscopic ovarian cystectomy
BS: Bilateral salpingectomy
BMI: Body mass index
TVUS: Transvaginal ultrasound
OR: Odds ratio
CI: Confidence interval
FHB: Foetal heart-beat
CRL: Crown-rump length
GS: Gestational sac
MRI: Magnetic resonance imaging
HCG: Human chorionic gonadotrophin
MTX: Methotrexate
FIGO: International Federation of Gynecology and Obstetrics
